# 

**Published:** 1851-07

**Authors:** 


					﻿BOOKS RECEIVED.
We have received from the publishers, Messrs. S. S. & W. Wood,
of New York, a copy of a little work on Menstruation, by Edw. John
Tilt, M. D., said to be one of the best ever published on that subject,
and also the valuable Lectures of Dr. George Gregory on Eruptive
Fevers, of which we shall take the earliest opportunity to give a full
notice.
Recfipis of bie/Hcat Journal for June, 1851.
Dr. Haggard, Gallatin, Tenn., -	•	-	-	$5	00
W. E Daily, Hustonville, Ky.	-	-	-	5	00
---- Davidson. Petersburg, Ind., -	-	-	5 00
G. P. Robinson. Qonnty, .	-	-	-	5	00
D. C. Ross. Lisbon. Ark., -	-	-	-	10	00
J. Roberts. Monttezuma, Ind.,	-	-	-	10	00
J. Lundy, .Salem, Ky., -	-	-	-	5 00
The Wks ie1-' Joins ai. ok	mid M'rgkk v is published monthly by
the nnilpr-tif-i.-tl at tne co> er ot .Main and t’lltli streets. Louisville, al $5 per
annum p i ■ mee : •> a i'. 'iik o Each mimbet columns !)(» pages, making two vol-
atile- in tie v.-ar nt more than pa»>*« each
Contritm i n•-1<> >t- pa.y- tn the I'livstcian.. of the Valley of the Mississippi,
add	to Editors, are re-peeltnilv solicited.
Letters uu b unucas, a» be addressed, postage paid, to the publishers.
PKEK1KJE A CO.
				

